# Finite Element Analysis of Three-Dimensional (3D) Auxetic Textile Composite under Compression

**DOI:** 10.3390/polym10040374

**Published:** 2018-03-27

**Authors:** Jifang Zeng, Hong Hu

**Affiliations:** Institute of Textile and Clothing, The Hong Kong Polytechnic University, Hung Hom, Hong Kong, China; zengjifang@gmail.com

**Keywords:** 3D textile auxetic composite, negative Poisson’s ratio, compression properties, finite element (FE) analysis

## Abstract

This paper reports a finite element (FE) analysis of three-dimensional (3D) auxetic textile composite by using commercial software ANSYS 15 under compression. The two-dimensional (2D) FE model was first developed and validated by experiment. Then, the validated model was used to evaluate effects of structural parameters and constituent material properties. For the comparison, 3D non-auxetic composite that was made with the same constituent materials and structural parameters, but with different yarn arrangement in the textile structure was also analyzed at the same time. The analysis results showed that the auxetic and non-auxetic composites have different compression behaviors and the auxetic composite has better the energy absorption capacity than the non-auxetic composite under the same compression stress. The study has provided us a guidance to design and fabricate auxetic composites with the required mechanical behavior by appropriately selecting structural parameters and constituent materials.

## 1. Introduction

Auxetic composites are a special type of materials with negative Poisson’s ratio (PR), which expand when stretched and vice versa. In addition to the properties of composite, auxetic composites also take the advantages of auxetic materials, such as enhanced indentation resistance [1], improved durability of fatigue [2,3], and improved energy absorption capability [3]. According to Alderson et al. [4], two approaches could be adopted for the fabrication of auxetic composites. The first one is using specially designed configurations with non-auxetic constituent materials (reinforcement and matrix) and the other is to include auxetic elements in a composite or use auxetic constituents, for example, auxetic matrix, auxetic reinforcement, or both [2,5,6].

With regard to the first approach, the composites fabricated with non-auxetic materials by using specially designed configurations are laminates [7,8,9,10]. Milton [11] theoretically proposed an auxetic composite that was generated by layering component materials in different directions on widely separated length scales and found that negative PR of composites could reach −1. Alderson et al. [12] found that the low velocity impact resistance of auxetic carbon fiber laminates is better than that of laminates with near zero and positive PR.

With regard to the second approach, Wei and Edwards [5], firstly, theoretically predicted existence of auxeticity windows for composite by randomly dispersed auxetic spheres into a non-auxetic matrix and verified by experiment. They found that the indentation resistance of the composite could be improved by enhancing the modulus of elastic matrix. But, the inclusion shape is not restricted to sphere. Yet, plenty of reinforcements have been proposed or developed to fabricate auxetic composites, such as specific two-dimensional (2D) triangle inclusions [13,14,15], three-dimensional (3D) tetrahedron hollow shell [16,17], cellular honeycomb structure [18,19], double arrowhead [20,21], and chiral network [22]. Hou et al. [14,15,16,17] found that the mechanical properties and deformation behavior of auxetic composites can be tailored by controlling the geometry features and the density of inclusions, and these findings were confirmed by Wang et al. [6], Hu [13], and Li et al. [18]. Recently, Bruggi et al. theoretically investigated the synthesis of auxetic structures by a topology optimization approach for micropolar materials. They proposed a design approach that was applied to elements composed by microstructured material with prescribed properties. This work successfully sketched auxetic structures and microsystems [23].

Different from the discrete inclusions of auxetic reinforcements, Ge et al. [24,25,26,27] proposed a novel 3D auxetic textile structure for composite reinforcement based on the modification of a 3D conventional orthogonal woven structure. Using a 3D unit cell, the auxetic behavior of the structure was experimentally verified [25], theoretically analyzed [24,25], and numerically studied [26,27]. Both of curved [24] and straight [25,26,27] weft yarns were considered as the initial configuration. By embedding this auxetic structure into zero PR polyurethane (PU) foam, the 3D auxetic composite was fabricated and its compression auxetic behavior was studied by Zhou et al. [2] experimentally. Meanwhile, a simplified equation and a 2D FE model were also developed to theoretically predict its negative PR behavior [28]. In addition, its low-velocity impact properties were recently investigated as well [29]. By using ABS tubes to replace the warp yarns and eliminating the stitch yarns, Jiang et al. [30] proved that the auxetic effect was not diminished in their 3D composite. Limited by the fabrication process, the samples of 3D auxetic composites used for experimental tests by Jiang [30] and Zhou [2] were very restricted. Due to this limitation, the compression properties, including energy absorption capability (EAC), as well as effects of the structural parameters and constituent materials have not been fully investigated. Hence, the compression properties of the 3D auxetic composite fabricated with the same 3D textile reinforcement structure, as proposed by Zhou [2], were further investigated by using finite element (FE) method in this study. It is expected that this study could make us better understand the mechanical behavior of this novel 3D auxetic composite and could provide us guidance in designing and fabricating auxetic composites with required mechanical properties by appropriately selecting geometrical parameters and constituent materials.

## 2. Methodology

### 2.1. Brief Introduction of 3D Auxetic Textile Composite

**Figure 1:**
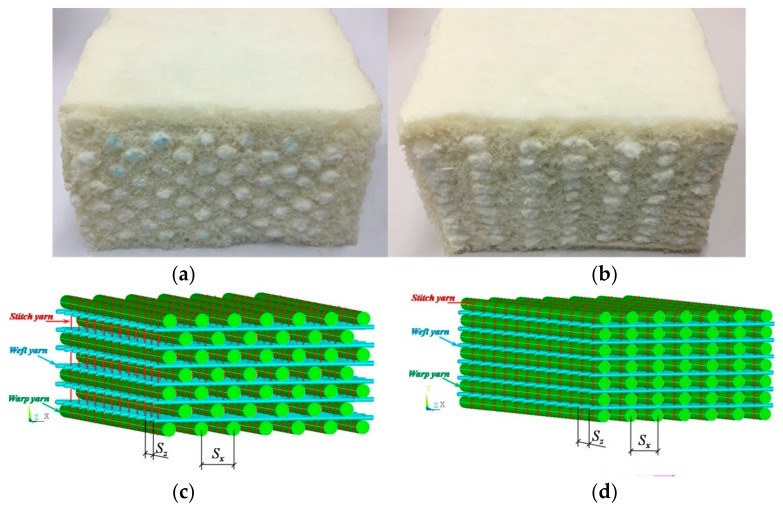
Three-dimensional (3D) textile composites and their reinforcement structures: (**a**,**c**) auxetic; (**b**,**d**) non-auxetic. Note: the composites shown in (**a**,**b**) are taken from Reference [29].

The 3D auxetic textile composite is shown in Figure 1a [29]. It is made with a specially designed 3D auxetic textile reinforcement structure (Figure 1c) and PU foam. As shown in Figure 1c, the auxetic reinforcement structure is formed by three yarn systems: warp yarns, weft yarns, and stitch yarns. Both the warp and weft yarns are used as reinforcing yarns. They are alternately arranged layer by layer into different configurations. While the warp yarns are arranged in a staggered form, the weft yarns are in a non-staggered form. The stitch yarns are used to bind the weft and war yarns together to form an integrated 3D textile structure. The use of the stitch yarns is not only to fix the weft and warp yarns, but also to avoid possible delamination of the structure. Due to the different weft and warp yarn arrangements, the weft yarns get corrugated under action of the adjacent warp yarns when the composite is compressed in *Y* direction, which leads to the shrinkage of the composite in *X* direction. As a result, auxetic effect is obtained in *XOY* plane of the composite. Differently, the auxetic behavior vanishes when the 3D auxetic composite is compressed in the *X* direction. With the increase of applied compression strain in *X* direction, the adjacent warp yarns get close to each other and the weft yarns buckle. In a similar reason, the auxetic behavior also cannot be achieved when they are compressed in *Z* direction. Therefore, only the compression behavior of the 3D auxetic composite in *Y* direction is studied in this work.

The 3D auxetic composite was fabricated via a special process. The weft and warp yarns were first placed into a mold and were bound together by stitch yarn to form a 3D auxetic textile reinforcement structure. Then, the chemical solution, which was uniformly mixed with polyether polyol and isocyanate, was injected into the mold. Finally, 3D auxetic textile composite was formed with the PU foam as matrix after a forming process. As the weft and warp yarns were fixed by the mold, they were kept into a straight form during the whole fabrication process. For the comparison, 3D non-auxetic composite that is made of the same constituent materials and structural parameters is also included in this study, as shown in Figure 1b [29]. The difference between the 3D auxetic and non-auxetic textile composites consists in the different arrangement of the warp yarns in XOY plane, as shown in Figure 1d. The details of fabrication process for the two types of composites can be found in Reference [2].

From Figure 1, it can be seen that the geometry of the composites can be determined by diameter of warp yarn *D*_1_, diameter of weft yarn *D*_2_, warp yarn spacing *S*_x_, weft yarn spacing *S*_z_, and composite lengths in *X*, *Y*, and *Z* direction.

### 2.2. Finite Element Modeling

**Figure 2:**
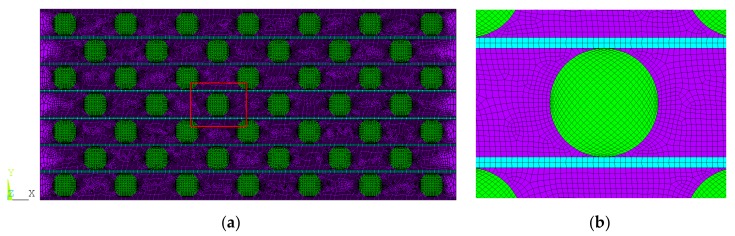
(**a**) The two-dimensional (2D) finite element (FE) model of 3D auxteic composite and (**b**) local amplified image marked by red square in (**a**). Note the warp yarns are colored in green, the weft yarns in cyan and PU foam in purple.

The FE modeling was conducted by using the commercial software ANSYS 15 (ANSYS, Inc., Pittsburgh, PA, USA). As mentioned before, the auxetic composite has only negative PR in XOY plane when compressed in *Y* direction. Therefore, the weft yarns in each layer can work together like a corrugated sheet under compression, as long as their spacing is small enough. Furthermore, the length of the warp yarns is much larger than the thickness of auxetic composite (the size in *Y* direction). In addition, the stitch yarns could be omitted in FE modeling, because they do not bear force under compression. From these considerations, the compression properties of auxetic composite could be simulated via a plane strain model, as shown in Figure 2a by assuming the warp and weft yarns to be straight in initial state. As the warp and weft yarns are very flexible and the PU foam is hyperelastic and soft, it is further assumed that the warp and weft yarns are bounded well with the PU foam and no crack occurred during compression. Therefore, no failure criteria are used in this simulation.

The ANSYS Parameter Design Language (APDL) was used to create the FE model. The geometrical parameters and mechanical properties of constituent materials were selected as modeling parameters. Based on the investigation on the size effect of FE model that is presented in Appendix A, the geometrical parameters listed in Table 1 were used to create the control model. The FE model was created from the bottom layer to the upper layer. In this case, the weft yarns, PU foam, and warp yarns were created successively. The detailed modeling process can be found in Ref. [28].

Meshing was carried out by using PLANE182 element type (ANSYS, Inc., Pittsburgh, PA, USA). Due to the fairly regular areas, the warp and weft yarns could accept a mapped mesh. To reduce the element number, only quadrilateral elements were used to mesh the warp and weft yarns. However, the PU foam had to be meshed freely due to its irregular geometrical areas. To clearly show the element shape, an amplified local image of the FE model is shown in Figure 2b. It can be seen that the regular pattern of elements of the warp and weft yarns greatly increased the quality of the mesh. Because the interfaces among warp yarns, weft yarns, and PU foam were simulated by sharing the same lines to ensure that the warp and weft yarns were bounded well in PU foam matrix, no contact elements were defined in the FE model.

For the boundary conditions, all nodes located in the bottom were fixed in *Y* direction and the nodes in the top were coupled together in the *Y* direction to simulate the compress test of the composite between the two compression plates. In the *X* direction, the nodes in four corners were fixed to make sure no translation occurred. The compression force was applied to the node in the top layer of the FE model. To facilitate the convergence, the displacement load was applied in this simulation. Meanwhile, the displacement load was also easy to control and calculate the compression strain in *Y* direction. Since no contact elements were used, the calculation efficiency was high, and the solution converged easily.

For the material properties, both the warp and weft yarns were simulated as transversal isotropic materials with Young’s modulus *E*_x_ in lengthwise direction, transversal modulus *E*_r_ in radial direction, and PR ν. To the best of our knowledge, the transversal modulus *E*_r_ of yarn was rarely reported. Meanwhile, the value of *E*_r_ was usually smaller than *E*_x_. Here, the ratio *E*_r_/*E*_x_ was 1/60 for control model and its effect would be carefully discussed in the section of results and discussion. To exactly simulate the compression behavior of the auxetic composite, the equivalent bending stiffness was used to simplify weft yarns, keeping their Young’s modulus unchanged. For the PU foam, the two-parameter Ogden model was used to simulate its hyperelastic behavior and the details of simulation are shown in Appendix B. The material properties of PU foam shown in the first row of Table A1 are used to create the control model in this work.

In the same way, the FE model for the non-auxetic composite with the same structural parameters and constituent materials properties was also created for comparison.

### 2.3. Calculation of Compression Behavior

**Figure 3:**
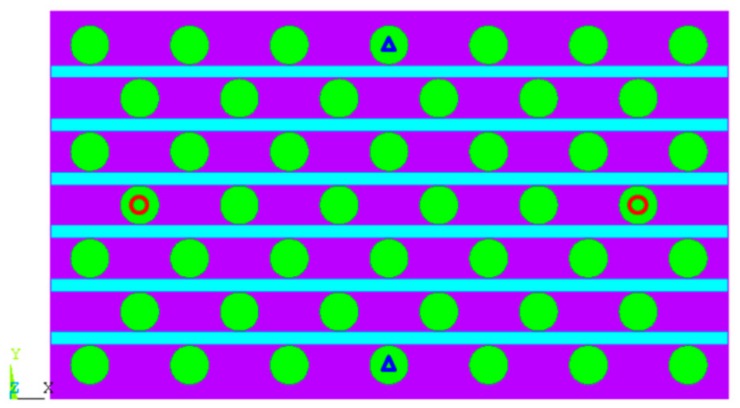
Illustration of warp yarns used for calculation of Poisson’s ratio.

Because the four corners were fixed to simulate the compression test of auxetic composite, the shrinkage for each yarn layer was varied on both the left and right edges. For this reason, the warp yarns that are located in the middle layer (marked by red circles in Figure 3) were suitable for the characterization of the contraction strain ε_x_ in the *X* direction to get rid of the edge effect. For the compression strain ε_y_ in the *Y* direction, it was calculated from the relative displacement of two warp yarns (blue triangles in Figure 3), divided by their original distance. When the contraction strain ε_x_ and compression strain ε_y_ are known, the PR of auxetic composite could be calculated from the equation $\nu =-\varepsilon_{x}/\varepsilon_{y}$.

Because true stress is not possible to adequately represent the instantaneous behavior of a highly strain dependent material, engineering stress, $\sigma =p/L_{x}$ was defined to characterize the compression resistance and called stress for short in this work, where, *p* is the compression force that was obtained by the reaction force at the bottom of the FE model, *L_x_* is the total length of FE model in *X* direction. From here, the EAC can be calculated by the integration of stress-strain curve in the form of $E=\int_{0}^{\varepsilon_{y}}\sigma d\varepsilon$.

## 3. Results and Discussion

### 3.1. Verification of FE Simulation

**Figure 4:**
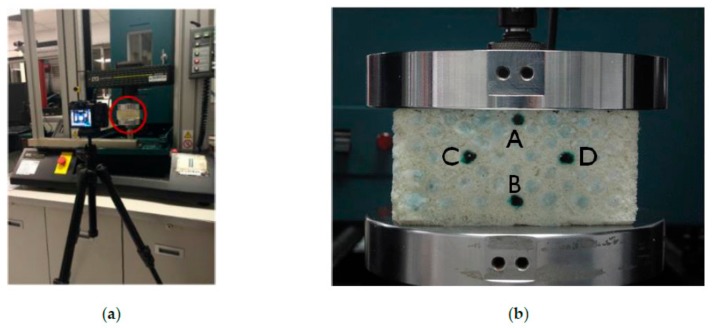
The compression test: (**a**) the experimental setup; (**b**) 3D auxetic composite placed between two circular plates. The black points marked on the cross section of the composite are used for the measurement of the distance changes in the vertical and horizontal direction. Note: (**a**,**b**) are redrawn from Ref. [2].

**Figure 5:**
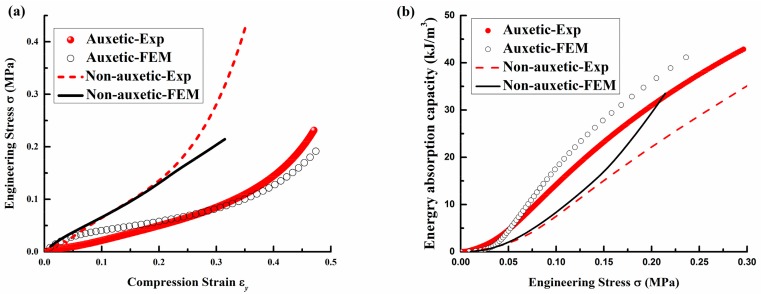
The results from experiment and finite element model (FEM) simulation: (**a**) strain-stress curve and (**b**) energy absorption capability (EAC)-stress curve.

The FE simulation is first verified by the compression results of both the auxetic and non-auxetic composites that were obtained in our previous study [2]. The compression test was conducted at a constant strain rate of 0.01/s on an Instron 5566 Universal Testing Machine (Instron Worldwide Headquarters, Norwood, MA, USA) using a 10 kN load cell. As shown in Figure 4a, a camera Canon PowerShot G10 (Canon, Tokyo, Japan) with a timer shot function was placed in front of the composite to take a picture of its cross-section every 2 s during the compression. As shown in Figure 4b, four warp yarns (numbered from A to D) of the composite were marked in black to facilitate the deformation measurement. Before the compression, a picture of the composite was taken firstly to measure the initial values of the vertical (A-B) and horizontal (C-D) distances. Then, the compression strain could be calculated by the changes in the vertical distance and the contraction strain is obtained by the changes in horizontal distance. The same constituent materials properties from the tested auxetic and non-auxetic composites, as listed in Table 2, were used for the FE simulation. Both the stress-strain and EAC-stress curves from the experimental and FE simulation results for both are shown in Figure 5a,b, respectively. From the Figure 5a, it can be seen that the stress is overestimated by the FE simulation at low strain for auxetic composite. The reason is that the Young’s Modulus is the Secant modulus, which is larger than its true modulus. But, for non-auxetic composite, the stress is underestimated by the FE simulation when the strain is more than 20%, because the buckling load may be increased by the stitch yarns, which are ignored in FE simulation. In Figure 5b, although the EAC is overestimated for the auxetic and non-auxetic composites by the FE simulation, the EAC of the auxetic composite is still higher than the EAC of the non-auxetic composite for both of the experimental and FE simulation results. Based on the above comparison, it can be confirmed that the FE models for both the auxetic and non-auxetic composites are suitable for characterizing their compression behavior. Therefore, the FE simulation results are used to discuss the typical compression behavior of auxetic composite and effects of structural parameters and constituent material properties in the following sections.

### 3.2. Typical Compression Behavior of Auxetic Composite

**Figure 6:**
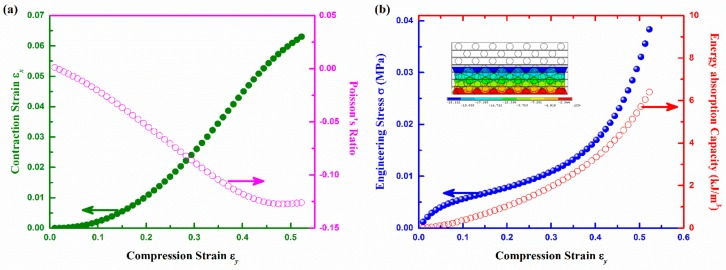
Strain dependent behavior of auxetic composite: (**a**) contraction strain and Poisson’s ratio (PR); (**b**) stress and EAC with inset of displacement contour at ε_y_ = 0.5 and undeformed edge.

As shown in Figure 6, the contraction strain ε_x_, PR, stress, and EAC as a function of compression strain ε_y_ calculated from 2D FE model were used to characterize the typical compression behavior of the auxetic composite. Form Figure 6a, it can be seen that both ε_x_ and negative PR effect increase with the increase of compression strain ε_y_ up to around 45%. After that, the negative PR effect slightly decreases due to the increased cross-sectional deformation of warp and weft yarns under high compression. From Figure 6b, it is found that the stress-strain curve could be divided to three regions with the increase of ε_y_. In the first region, the stress increases quickly before ε_y_ reaches 5%. After that, the stress raises gradually until ε_y_ reaches 40%. A large deformation in this region indicates an excellent EAC of the auxetic composite. Meanwhile, the weft yarns get crimped and the warp yarns are moved inward, resulting in an enhanced densification and compression resistance. From the inset of Figure 6b, it is suggested that the PU foam between two weft yarns is compressed to be very thin. Then, in the third region, the stress dramatically increases until some elements distort seriously and the solution is not convergent. The curvature of weft yarn also reaches its maximum value. From Figure 6b, it is found that the EAC increases with ε_y_ in a power form.

**Figure 7:**
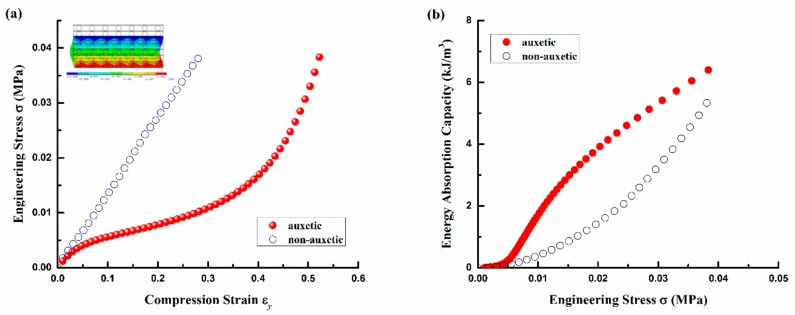
Comparison of auxetic and non-auxetic composite: (**a**) stress-strain curve; (**b**) EAC and stress. Note: the inset is the displacement contour at ε_y_ = 0.3 and undeformed edge for non-auxetic composite.

Figure 7 shows a comparison between the auxetic and non-auxetic composites made with the same structural parameters and constituent materials properties. As shown in Figure 7a, the stress-strain curve of the non-auxetic composite is nearly a straight line, which is very different from that of the auxetic composite. The different shapes of the stress-strain curves between the auxetic and non-auxetic composites indicate that they have different compression behaviors. While the auxetic composite behaves as a softer material, the non-auxetic composite behaves as a stiffer material.

In order to reveal which composite could absorb more energy under the same compression stress, the EAC as a function of compression stress instead of ε_y_ is shown in Figure 7b. It is clearly shown that the auxetic composite can absorbs more energy than the non-auxetic composite under the same compression stress. However, when the stress exceeds 0.04 MPa, the difference in the EAC between two types of composites is getting smaller. The reason is that the auxetic composite gets more compact with the increase of ε_y_. When ε_y_ exceeds 45%, the difference between the auxetic and non-auxetic composites becomes smaller due to the compacting of the composite structure.

The difference in compression behavior of the auxetic and non-auxetic composites mainly comes from the different arrangement of warp yarns in their textile reinforcement structure. In the 3D auxetic structure (Figure 1c), the warp yarns are alternatively stacked, which can cause a larger compression deformation of the composite. Conversely, in the non-auxetic structure (Figure 1d), the warp yarns are arranged in the vertical lines, which can withstand a higher compression load, but a lower deformation strain under compression. This is why the non-auxetic composite absorbs less energy under the same compression stress than the auxetic composite. Buckling is another problem of the non-auxetic structure (see inset in Figure 7a), which could result in the collapse of the structure under high compression strain.

### 3.3. Effect of Geometrical Parameters of 3D Textile Composites

As mentioned before, the geometry of FE model is determined by diameter of warp yarn *D*_1_, diameter of weft yarn *D*_2_ and warp yarn spacing *S*_x_. In order to investigate effects of these structural parameters on the compression behavior of 3D auxetic and non-auxetic textile composites, two aspect ratios ξ_1_ and ξ_2_ defined as ξ1=SxD1,ξ2=D2D1 were introduced. Here, *D*_1_ is used to normalize *D*_2_ and *S*_x_. Obviously, *D*_2_ and *S*_x_ can also be used for normalization.

**Figure 8:**
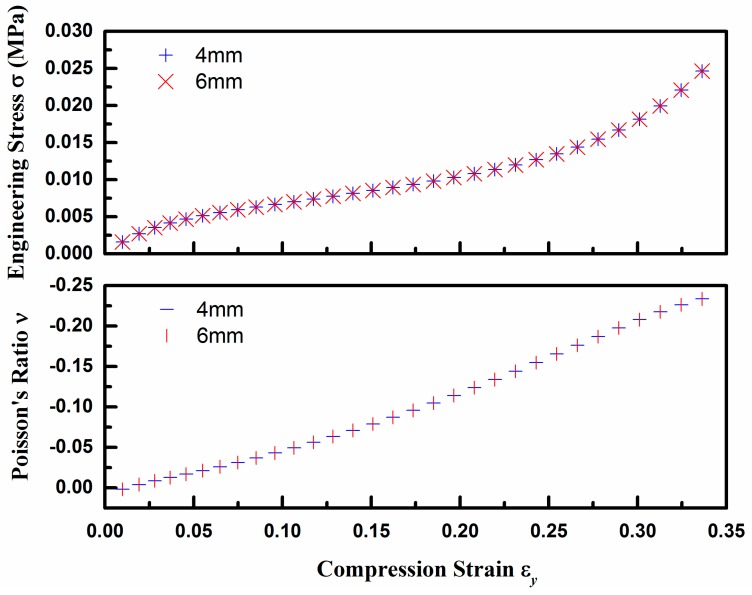
The stress and PR of auxetic composite for given ξ_1_ = 2.0 and ξ_2_ = 0.1.

To verify the rationality of this normalization, two different values *D*_1_ = 4 mm and 6 mm were first selected for comparison for given ξ_1_ = 2.0 and ξ_2_ = 0.1 as an example. The calculated stress and PR as a function of ε_y_ are shown in Figure 8. No difference is observed between 4 mm and 6 mm warp yarns for both of stress and PR, which indicates that the compression properties of auxetic composites are determined by the aspect ratios among the structural parameters, not by material scale.

**Figure 9:**
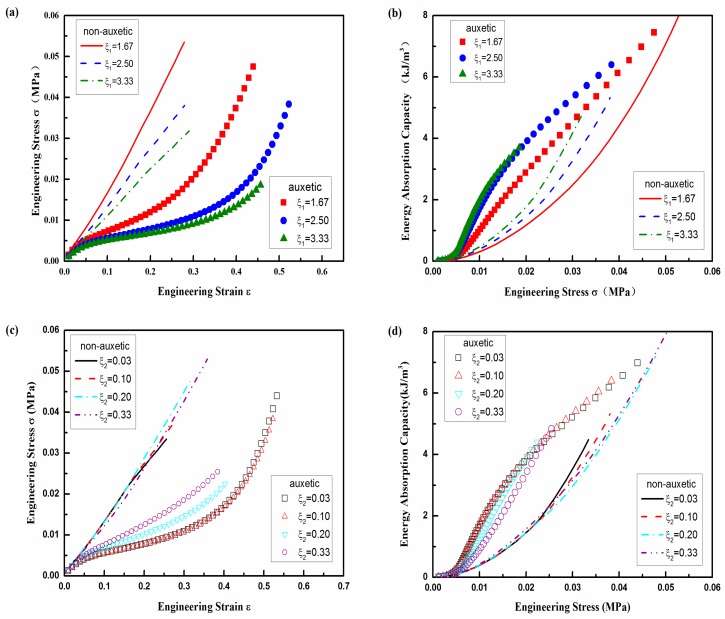
Geometrical effects: (**a**) stress-strain curve and (**b**) EAC vs. stress for ξ_1_; (**c**) stress-strain curve and (**d**) EAC vs. stress for ξ_2_.

Fixing all the other parameters, the effects of ξ_1_ on compression behavior of auxetic and non-auxetic composites are displayed in Figure 9a,b, respectively. From Figure 9a, it can be seen that the stress decreases with the increase of aspect ratio ξ_1_ for both the auxetic and non-auxetic composites. This is normal because the increase of ξ_1_ also increases the spacing between the warp yarns, resulting in a capacity decrease of reinforcement structure to withstand the compression load. But, the difference in stress is very small when ξ_1_ changes from 2.50 to 3.33 for the auxetic composite. The similar situation is also observed for EAC. As shown in Figure 9b, the difference of EAC gets negligible when ξ_1_ changes from 2.50 to 3.33. It means that there is a limitation on the increase of EAC by only increasing warp yarn spacing. Accordingly, ξ_1_ = 2.50 is an excellent geometry when ξ_2_ is 0.1 for the auxetic composite.

The effects of ξ_2_ are shown in Figure 9c,d respectively, when all the other parameters are fixed. From Figure 9c, it can be seen that ξ_2_ does not have obvious effect on stress of the non-auxetic composite, but have an obvious effect on that of the auxetic composite. The similar phenomenon is also observed for the EAC, as shown in Figure 9d. This difference in ξ_2_ effect between the auxetic and non-auxetic composites is due to the arrangement of the warp yarn. As mentioned before, the warp yarns are arranged in the forms of vertical lines in non-auxetic composite. With this arrangement, the compression behavior of the non-auxetic composite mainly depends on the radial compression properties of warp and weft yarns, not their ratio. However, the increase of ξ_2_ may increase the buckling trend of the non-auxetic composite, which causes a small variation of the compression properties of the non-auxetic composite at higher compression strain or stress. For the auxetic composite, the compression stress increases with the increase of ξ_2_ due to the increase of the bending stiffness of the weft yarns originated from the increase of the weft yarn diameter. However, the EAC has a decreasing trend with the increase of ξ_2_ as the weft yarns get stiffer and ε_y_ decreases under the same compression stress. The results suggest that a lower ξ_2_ is required if a higher EAC is wanted. However, the decrease of ξ_2_ results in the lower compression resistance. For the balance between the stress and EAC, a suitable ξ_2_ should be selected according to the final application. For the all above cases, the auxetic composite absorbs more energy than its counterpart non-auxetic composite under the same stress, which indicates that the auxetic composite is a better energy absorber.

### 3.4. Effect of Yarns Properties

As mentioned before, the yarns were assumed as transversally isotropic materials with Young’s modulus *E*_x_ in lengthwise direction and transversal modulus *E*_r_ in radial direction. To facilitate the investigation of the effect of these moduli, two normalized moduli $E^{*}=E_{x}^{warp}/E_{x}^{weft}$ and $E^{\prime }=E_{r}^{weft}/E_{x}^{weft}$ were introduced.

**Figure 10:**
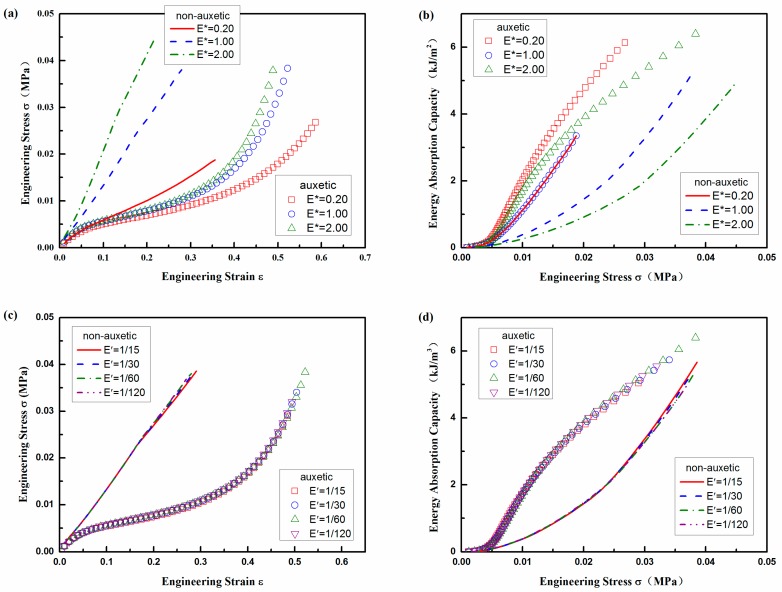
Material property effect of yarns: (**a**) stress-strain curve and (**b**) EAC vs. stress for E*; (**c**) stress-strain curve and (**d**) EAC vs. stress for *E*ʹ.

The effects of E* on the stress and EAC are shown in Figure 10a,b, respectively, when all the other parameters are kept unchanged. It can be seen that for both the auxetic and non-auxetic composites, the stress is increased with the increase of E* for the same compression strain (Figure 10a), but the EAC is decreased with the increase of E* for the same compression stress (Figure 10b). In addition, the effects of E* for the non-auxetic composite are more significant than those for the auxetic composite, indicating that the compression properties of the non-auxetic composite are more affected by the properties of the warp yarn. For the auxetic composite, as the compression strain mainly comes from the deformation of PU foam at beginning of compression, the effect of E* is not obvious at low compression strain and stress. However, with the increase of strain and stress, the PU foam is compacted and the cross-sectional deformation of the warp yarns increase. As a result, the effect of E* increases at high compression strain and stress. The results suggest that to get higher EAC under the same stress, a lower E* is required, which means that softer warp yarns should be used.

The effects of *E*′ on compression stress and EAC for both the auxetic and non-auxetic composites are illustrated in Figure 10c,d, respectively, when all of the other parameters are kept unchanged. It can be seen that the influence of ratio *E*′ is nearly negligible when the *E*′ increases from 1/120 to 1/15 (a range for most of ordinary yarns). The reason is that the main deformation mode of weft yarns in the auxetic composites is bending, which mainly depends on *E*_x_ in lengthwise direction, not *E*_r_ in radial direction. The same as aspect ratio’s effect, the auxetic composite absorbs more energy than its counterpart non-auxetic composite under the same given stress.

### 3.5. Effects of PU Foam Properties

**Figure 11:**
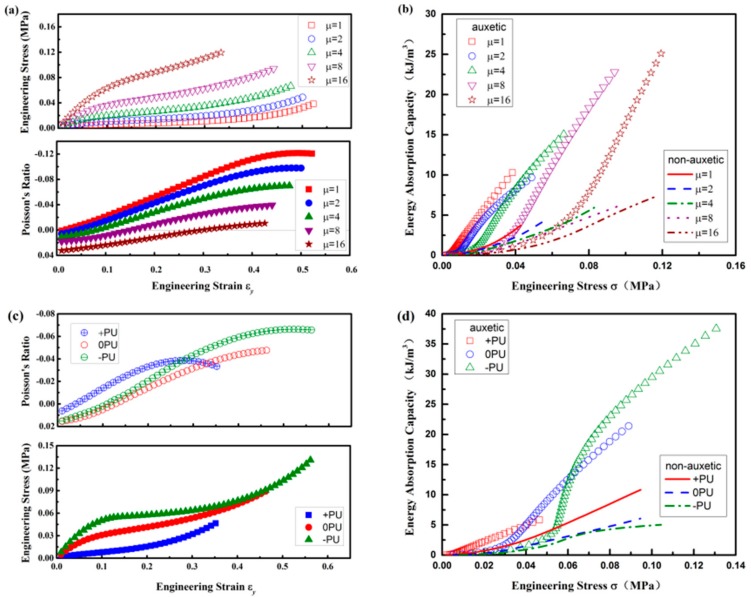
Effects of polyurethane (PU) foams: (**a**,**b**) with zero PR but with different initial shear moduli; (**c**,**d**) with different PRs. ((**a**,**c**) stress-strain and PR-strain curves of auxetic composites; (**b**,**d**) EAC-stress curves of auxetic and non-auxetic composites).

In this study, the PU foam was firstly simulated based on the Ogden model that possesses hyper-elastic behavior with zero PR. In order to investigate effects of PU foam properties on the compression properties of composites, two groups of PU foams were selected for analysis. The detailed descriptions of these PU foams are shown in the Appendix B. In the first group, all of the PU foams keep zero PR, but the initial shear moduli are changed. The PU foams with different initial shear moduli are indicated by μ. As shown in Figure 11a, the stress of the auxetic composite increases with the increase of μ, but the negative PR effect decreases with the increase of μ. When μ = 16, the PR of the composite approaches zero and the auxetic effect becomes insignificant. The main reason is that the PU foam gets stiffer with the increase of μ and is more difficult to be compressed by the crimped weft yarns. As a result, the EAC of the auxetic composite also decreases with the increase of μ due to lower deformation under the same stress, as shown in Figure 11b. Hence, the simulation results suggest that softer PU foam is more suitable for the fabrication of auxetic composite with higher EAC. In the second group, the PRs of PU foams are no longer kept unchanged and have positive PR (+PU), zero PR (0PU), and negative PR (−PU), respectively. As shown in Figure 11c, auxetic effect disappears when the strain is smaller than 10% for the auxetic composites fabricated with both the zero and negative PR PU foams. But their auxetic effect increases with the increase of strain and gets more remarkable than that of the auxetic composite fabricated with positive PR PU foam. Meanwhile, a wider plateau in stress-strain curve is observed for the auxetic composite that was fabricated with negative PR PU foam, indicating an excellent EAC. However, as shown in Figure 11d, the EAC of the auxetic composite fabricated with negative PR PU foam is lower than that of the composites fabricated with zero and positive PR foams at lower compression stress. The reason is that with the negative PR, the PU foam is easily compressed at lower compression stress which results in lower deformation. As a result, the EAC is not high. The results show that, although the auxetic composite fabricated with negative PR foams can absorb more energy under high stress, it is not suitable for the protection of very brittle objects. When considering the convenience in the fabrication of zero PR PU foam, auxetic composites that were fabricated with zero PR PU foam can be a great candidate for the protective application.

## 4. Conclusions

The compression properties of auxetic composite under the quasi-static compression were systematically investigated by using FE method and compared with those of non-auxetic composite. The effects of structural parameters and constituent material properties were analyzed and discussed. Based on the analyses, the conclusions are drawn as follows:

1. Auxetic and non-auxetic composites have different compression behaviors due to different yarn arrangements in their 3D textile reinforcement structure. While the auxetic composite behaves as a softer material, the non-auxetic composite behaves as a stiffer material. The auxetic composite has much better EAC than the non-auxetic composite made with the same structural parameters and constituent material properties.
2. All of the structural parameters and constituent material properties have different effects on the compression properties of auxtic and non-auxetic composites. For geometry effects, the decrease of ξ_1_ and increase of ξ_2_ can result in the increase of the compression stress and decrease of the EAC of the auxetic composite. For yarn property effects, the increase of E* can cause the increase of the compression stress and the decrease of the EAC of auxetic composite, but *E*′ has no effect on these properties. For PU foam effects, the increase of its initial shear modulus can result in the increase of the compression stress and the decrease of both EAC and auxetic behavior of auxetic composite.
3. The effects of PR depend on the level of compression strain and stress. At low compression strain and stress, changing PR of PU foam from positive to negative can cause the increase of the compression stress, but the decrease of both EAC and auxetic effect of auxetic composite. At high compression strain and stress, the effects of PR of PU foam are just opposite. Thus, the compression properties of auxetic composites can be tailored by proper selecting structural parameters and constituent material properties.

## Figures and Tables

**Table 1:** Structural parameters of the auxetic and non-auxetic composites (unit: mm).

*D*_1_	*D*_2_	*S*_x_	*L*_x_	*L*_y_
6	0.6	15	102	46.8

Note: the *L*_x_ and *L*_y_ are the length of composites in *X* and *Y* direction, respectively.

**Table 2:** Constituent materials properties for the auxetic and non-auxetic composites.

Warp Yarn		Weft Yarn		PU Foam					
*E*_x_ (MPa)	υ	*E*_x_ (MPa)	υ	μ_1_ (kPa)	μ_2_ (kPa)	α_1_	α_2_	β_1_	β_2_
30	0.2	30	0.2	16.8	12.25	19.8	19.7	1.4 × 10^−3^	6.6 × 10^−4^

**Figure A1:**
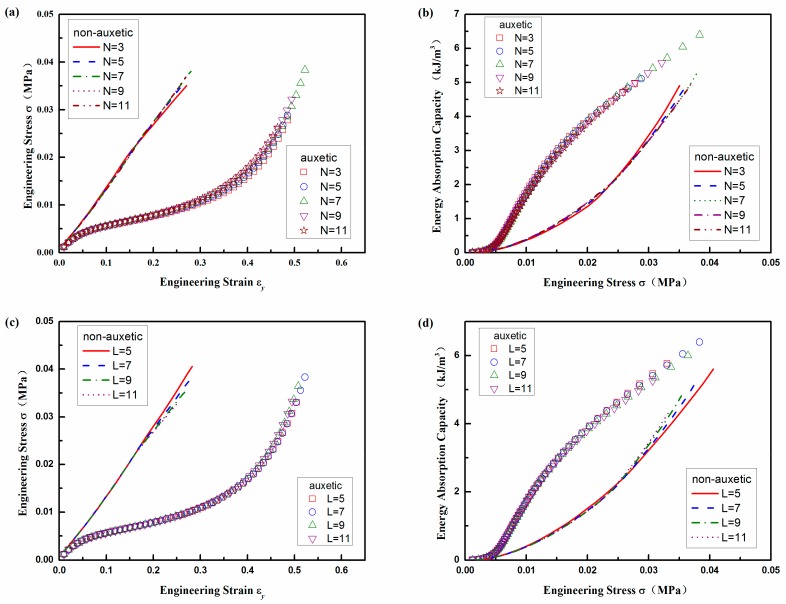
Size effect of FE model: (**a**) stress-strain curve and (**b**) EAC vs. stress for *N*; (**c**) stress-strain curve and (**d**) EAC vs. stress for *L*.

**Figure A2:**
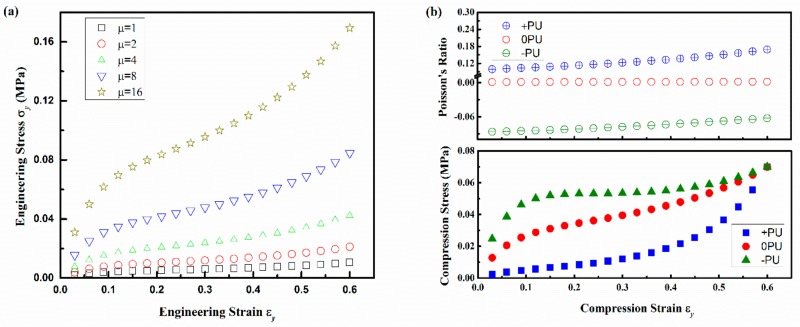
The constitutive relation for PU foam under uniaxial compression: (**a**) for Group 1 and (**b**) for Group 2 named by the sign of their PR as +PU, 0PU and −PU.

## Appendix A. Size Effect of Finite Element Model

The FE model size effect was studied to get rid of edge effect on simulation results under quasi-static compression, which is described by number of warp yarns in bottom layer *N* (Figure A1a,b) and total number of warp yarn layers *L* (Figure A1c,d), using the structural parameters and constituent materials properties listed in Table 1 and Table 2. From Figure A1, it can be seen that both *N* and *L* have no effect on compression properties of auxetic composite so that the control model is suitable for analysis.

## Appendix B. Mechanical Behaviors of PU Foam

In this study, non-linear stress-strain behavior of hyperelastic PU foam is accurately described by the Ogden material model with 2 parameters, based on the principal stretches of the left Cauchy-Green tensor. There are two groups of PU foams to be studied as listed in Table A1 and Figure A2, while the PR of PU foam in Group 1 nearly equals to zero.

**Table A1:** The material constants of PU foam.

Group	Definition	μ_1_ (kPa)	μ_2_ (kPa)	α_1_	α_2_	β_1_	β_2_
Group 1	μ = 1	2.4	1.8	19.8	19.7	1.4 × 10^−3^	6.6 × 10^−4^
	μ = 2	4.8	3.5	19.8	19.7	1.4 × 10^−3^	6.6 × 10^−4^
	μ = 4	9.6	7.0	19.8	19.7	1.4 × 10^−3^	6.6 × 10^−4^
	μ = 8	19.2	14.0	19.8	19.7	1.4 × 10^−3^	6.6 × 10^−4^
	μ = 16	38.4	28.0	19.8	19.7	1.4 × 10^−3^	6.6 × 10^−4^
Group 2	+PU	2.4	1.8	19.8	19.7	1.4 × 10^−1^	6.6 × 10^−2^
	0PU	15.8	11.6	19.8	19.7	1.4 × 10^−3^	6.6 × 10^−4^
	−PU	34.5	25.2	19.8	19.7	−1.4 × 10^−1^	6.6 × 10^−4^
